# Small Molecule KRAS Inhibitors: The Future for Targeted Pancreatic Cancer Therapy?

**DOI:** 10.3390/cancers12051341

**Published:** 2020-05-24

**Authors:** Josef Gillson, Yogambha Ramaswamy, Gurvinder Singh, Alemayehu A. Gorfe, Nick Pavlakis, Jaswinder Samra, Anubhav Mittal, Sumit Sahni

**Affiliations:** 1 Northern Clinical School, Faculty of Medicine and Health, University of Sydney, St Leonards 2065, NSW, Australia; jgil9462@uni.sydney.edu.au (J.G.); nick.pavlakis@sydney.edu.au (N.P.); jas.samra@bigpond.com (J.S.); anubhav@mittal.com.au (A.M.); 2 Bill Walsh Translational Cancer Research Laboratory, Kolling Institute of Medical Research, University of Sydney, St Leonards 2065, NSW, Australia; 3 Australian Pancreatic Centre, St Leonards 2065, NSW, Australia; 4 School of Biomedical Engineering, Faculty of Engineering, The University of Sydney 2006, Sydney, Australia; yogambha.ramaswamy@sydney.edu.au (Y.R.); gurvinder.singh@sydney.edu.au (G.S.); 5 Department of Integrative Biology and Pharmacology, University of Texas Health Science Center Houston, Houston, TX 77030, USA; Alemayehu.G.Abebe@uth.tmc.edu; 6 Northern Sydney Cancer Center, Royal North Shore Hospital, St Leonards 2065, NSW, Australia; 7 Genesis Care, St Leonards and Frenchs Forest 2065, NSW, Australia; 8 Upper GI Surgical Unit, Royal North Shore Hospital and North Shore Private Hospital, St Leonards 2065, NSW, Australia

**Keywords:** pancreatic ductal adenocarcinoma, KRAS inhibitors, targeted therapies, drug delivery, nanoparticles, anti-fibrotic therapies

## Abstract

Pancreatic ductal adenocarcinoma (PDAC) is one of the deadliest solid tumors in the world. Currently, there are no approved targeted therapies for PDAC. Mutations in *Kirsten rat sarcoma viral oncogene homologue* (*KRAS*) are known to be a major driver of PDAC progression, but it was considered an undruggable target until recently. Moreover, PDAC also suffers from drug delivery issues due to the highly fibrotic tumor microenvironment. In this perspective, we provide an overview of recent developments in targeting mutant KRAS and strategies to overcome drug delivery issues (e.g., nanoparticle delivery). Overall, we propose that the antitumor effects from novel KRAS inhibitors along with strategies to overcome drug delivery issues could be a new therapeutic way forward in PDAC.

## 1. Introduction

Pancreatic cancer is the seventh most common cancer type globally and features an incidence to mortality ratio of approximately one [1,2,3]. Alongside an immovable 5-year survival rate of 6–10% [2], these dismal statistics makes pancreatic cancer one of the most difficult diseases to manage in modern medicine. Of all pancreatic cancer types, 93% are ductal adenocarcinomas (PDAC). The main lethality behind PDAC is the lack of early stage symptoms, resulting in disease progression to an advanced metastatic stage at diagnosis. Extensive research into PDAC has been done, but it is yet to yield a major breakthrough. While the scientific community continues to unearth the molecular mechanisms involved in progression of PDAC, we still do not possess the therapeutic tools to treat it by targeting these complex pathways. 

Currently, curative intent treatment for PDAC patients is limited to surgical removal of the tumor [4]. However, of all the patients diagnosed with PDAC, only 20% of patients are deemed resectable [4]. Thus, the majority of PDAC cases are managed palliatively by chemotherapeutic approaches. The most commonly used therapeutic regimens against PDAC involve gemcitabine and 5-fluorouracil (5-FU), both antimetabolites, which exhibit their antitumor effects by inhibiting DNA synthesis, leading to the death of rapidly dividing tumor cells. However, recent “success” with chemotherapy has been attained mainly through combination therapy since pancreatic tumors tend to be highly resistant to chemotherapy. The large randomized phase III study by Von Hoff et al. [5] compared the combination of gemcitabine and *nab*-paclitaxel (*nab*-P/G) to gemcitabine alone (G), demonstrating an improved tumor response rate (RR) to 23%, and overall survival (OS), with 35% in the *nab*-P/G group versus 22% in the G group alive at 1 year, and 9% versus 4% at 2 years, respectively. Similarly, Conroy et al. [6] compared modified FOLFIRINOX (combination therapy of folinic acid, 5-FU, Irinotecan and Oxaliplatin) to G monotherapy, also observing an improved RR to 31.6%, and a 43% improvement in the survival rate (OS hazard ratio 0.57, *p* < 0.001), but at the cost of a significant increase in toxicity and a corresponding detriment in quality of life. Both these regimens were adopted as significant advances, and thus went on to be evaluated in the adjuvant settings. Here, Conroy et al. [7] demonstrated an improved OS rate at 3 years of 63.4% with adjuvant modified-FOLFIRINOX compared with 48.6% with G alone (OS HR 0.64, *p* = 0.003), but at the expense of greater toxicity, 75.9 % versus 52.9%, respectively. Additionally, in the adjuvant setting, in the APACT trial [8], *nab*-P/G failed to show an increase in the primary endpoint of investigator-assessed disease-free survival, but the interim OS was 40.5 months (*nab*-P/G) versus 36.2 months (G) (HR, 0.82; 95% CI, 0.680–0.996; *p* = 0.045).

While these improvements are positive steps forward, the survival period and rates are only marginally extended for the toxicity trade-offs. Moreover, the “sledgehammer” approach of adjuvant combination chemotherapy (applicable to FOLFIRINOX and to a lesser extent *nab*-P/G) can only be applied to highly selected fit patients following curative surgery. PDAC is thus still widely considered a chemotherapy-resistant disease where urgent high impact therapeutic breakthroughs applicable to broader patient populations are required. 

## 2. KRAS Inhibitors as Novel Anti-Cancer Agents for PDAC

PDAC is known to be a highly aggressive disease that usually features a highly mutated genetic landscape. The most commonly mutated genes in PDAC includes *Kirsten rat sarcoma viral oncogene homologue* (*KRAS*), *cyclin-dependent kinase inhibitor 2A* (*CDKN2A*), *tumor protein 53* (*TP53*), and *mothers against DPP homolog 4* (*SMAD4*) [9]. A specific oncogenic mutation of *KRAS* is known to be the first mutational change leading to the initiation of precancerous lesions in the pancreas [10,11]. In its wild-type state, KRAS is a membrane-bound GTPase that relays growth signals to the nucleus via the MAPK and PI3K pathways through GTP binding [12]. Activated receptor tyrosine kinases (RTKs), like epithelial growth factor receptor (EGFR), recruit guanine nucleotide exchange factors (GEFs) to promote the exchange of KRAS-bound GDP into GTP [13]. Oncogenic KRAS mutants remain bound to GTP in an active state and continue to perpetually exert cellular growth signaling [14]. 

*KRAS* is widely considered as one of the most frequently mutated oncogenes across all neoplasms (~30%) and appears in over 90% of PDAC [12]. It most frequently mutates at codon 12 (i.e., G12A/C/D/F/L/R/S/V), which accounts for 98% of mutations, with G12D being the most common (51%), followed by G12V (30%), G12A/C/S (2% each), and G12L/F (<1%) [15]. In addition, less frequent (<1%) mutations are also observed at codon 13 (i.e., G13C/D/P/S) and codon 61 (Q61H/K/R) [15]. A recent study demonstrated a strong selective pressure in the acquisition of the mutant *KRAS* allele in a PDAC mouse model, with two thirds of cancers demonstrating amplification of the mutant *KRAS^G12D^* allele [16]. Moreover, allelic gain of mutant *KRAS^G12D^* also correlated with an associated loss of the *KRAS^WT^* and increased metastatic potential [16].

KRAS is a 21-kDa protein with a nucleotide-binding site that is unsuitable for competitive inhibition and lacks deep hydrophobic pockets for allosteric inhibitor binding [15,17]. Interestingly, studies have also elucidated transient pockets on the protein surface, which might be suitable for the binding of small molecule inhibitors [18,19,20]. Recently, Marín-Ramos et al. [21] reviewed the KRAS inhibition strategies developed over the past four decades, where they highlighted that the struggle for an effective inhibitor stems from the inability of agents to effectively and efficaciously bind to small binding pockets, coupled with a highly competitive GTP concentration, rendering the KRAS protein undruggable. This problem inspired other possible indirect inhibitory routes involving the downstream targets of KRAS: RAF, MEK, and subsequently ERK. However, MAPK pathway inhibitors found an unexpected upregulation of the corresponding PI3K pathway, lacked efficacy, and exhibited high toxicity [22,23]. Currently, there are no KRAS inhibitors that have made it past early phase clinical trials. As such a common contributor to PDAC pathogenesis, it is understandable why inhibition of KRAS or these pathways is deemed as a priority. 

Recently, there have been some breakthroughs that have exploited KRAS as a therapeutic target, with promising prospects of developing a novel therapeutic approach for PDAC treatment. In 2018, a structure-based design study by Janes et al. [24] on the KRAS^G12C^ S-IIP-binding site pioneered and reinvigorated the search for KRAS inhibition through the discovery of a covalent compound, ARS-1620. This study demonstrated both in vitro efficacy and in vivo tumor regression from ARS-1620 and revealed novel KRAS inhibition. This result has refocused the spotlight back on KRAS as a potential druggable target and inspired the search for a much-desired breakthrough. Furthermore, Canon et al. [25] expanded the success of ARS 1620 to develop the superior compound named AMG 510, which was structurally very similar to ARS-1620. AMG 510 utilized the irreversible occupation of the His95 groove near the cysteine pocket to maintain a high level of inactive KRAS. The extensive study investigated in vitro, in vivo, and clinical assessments of this novel compound. They observed an almost complete inhibition of KRAS^G12C^ and subsequent downstream MAPK signaling in metastatic pancreatic and lung cancer cell lines (Mia-PaCa and NCI-H358). AMG 510 does not bind to wild-type KRAS or alter the cell viability of non-KRAS^G12C^ mutated tumors, indicating that it would be selective for mutant KRAS^G12C^ tumors. Following this initial success, the in vivo efficacy of AMG 510, either alone or in combination with PD 0325901 (MEK inhibitor) or carboplatin, was assessed using xenograft or syngeneic mouse models. Significant tumor regression was observed with AMG 510 alone, with a more potent effect demonstrated with combination therapy. The researchers, using a syngeneic mouse model, then incorporated a programmed cell death-1 antibody to block the immune checkpoint and demonstrated that 90% of the mice experienced a complete tumor regression. The preliminary clinical studies included four patients half responded with a >50% tumor shrinkage and one of them achieved a complete response. This small clinical trial provided supporting evidence to demonstrate the power of KRAS inhibition and the potential for compounds like AMG 510 to be developed further. These encouraging results have led AMGEN to initiate a phase I/II clinical trial involving 533 participants with KRAS^G12C^ mutations suffering from various advanced solid tumors, investigating the efficacy of AMG 510 as monotherapy or in combination with an anti-PD-1 immune checkpoint blocker. The initial results from this clinical trial are promising, with all patients (locally advanced or metastatic NSCLC patients treated with multiple failed prior therapies) treated at the highest/target dose (960 mg) achieving disease control (54% demonstrating partial response and 46% demonstrating stable disease) [26]. Only 35.3% of patients reported treatment-related adverse events, which were predominantly grade 1 and 2 nausea and vomiting [26]. Although these results are encouraging for cancers that majorly harbor KRAS^G12C^ mutations (e.g., lung cancers [27]), their utility for PDAC is limited, with only ~2% KRAS^G12C^ mutation observed [15]. Nonetheless, these studies will stimulate similar drug discovery initiatives targeting KRAS^G12D^ and KRAS^G12V^ mutations, which constitute ~80% of the KRAS mutations in PDAC [15].

It should also be mentioned that AMG 510 in the Canon et al. study exhibited EGFR accumulation upon its inhibition of KRAS. This observation was in direct concordance with previous studies [24,28,29], where MEK inhibitors potentiated HGF- and EGF-induced AKT pathway upregulation. It is understood that this is a compensatory homeostatic mechanism to sustain growth signaling when cells are subjected to MAPK interference. As this interaction has been observed across multiple studies, it must be investigated further. If not managed effectively, this counter mechanism could diminish the efficacy of KRAS inhibitors. To overcome this clinical issue, MAPK and PI3K/AKT inhibitors have been combined but also shown to have consistently reported severe toxicity [30,31], and hence, this is not considered as an alternative option currently. For instance, a randomized phase II trial compared the combination of Selumetinib (MEK inhibitor) and MK-2206 (AKT inhibitor) to modified FOLFIRINOX as a second-line therapy for metastatic PDAC patients [32]. Unfortunately, their efficacy was inferior to FOLFIRONIX and the toxicity greater [32]. Currently, there are approximately 10 ongoing clinical trials for MEK1/2 inhibitors (selumetinib, cobimetinib, and trametinib) for PDAC and it will be important to assess their outcomes before considering the clinical utility of MEK1/2 inhibitors for PDAC.

The adaptive response to KRAS inhibition, either by direct inhibition of mutant KRAS or via inhibition of the downstream signaling, is also shown to be mediated through upregulation of the pro-survival autophagic pathway [33,34]. Interestingly, studies have demonstrated the synergistic potential of KRAS- and autophagy-inhibitors in a variety of PDAC models [33,34]. Other mechanisms of adaptive resistance response to mutant KRAS inhibition have been discovered recently, such as the production of new mutant KRAS^G12C^, reactivation of the MAPK pathway, and inability to inhibit the PI3K-AKT pathway [35,36,37,38]. A recent study also identified collateral dependencies upon KRAS^G12C^ inhibition, which can be simultaneously targeted to enhance the susceptibility of tumors to direct KRAS^G12C^ inhibition [39]. Future clinical studies, utilizing combination studies of mutant KRAS inhibitors with inhibitors of these adaptive response mechanisms [35,36,37,38,39], will hold potential for the development of a potent and effective therapy for cancers harboring KRAS mutations.

Interestingly, AMG 510 is not the only novel KRAS inhibitor that has displayed clinical potential that has surfaced in the past couple of years. In 2019, a study by McCarthy et al. [40] also discovered a small molecule KRAS inhibitor, Compound 11, using a structure-based drug design approach and a variety of cellular and biophysical assays. Compound 11 is an allosteric pyrazolopyrimidine-based inhibitor that displayed high, sub-micromolar, affinity binding to an allosteric p1 pocket of both wild-type and mutant KRAS subtypes but not HRAS or NRAS. Compound 11 significantly disrupted KRAS signaling with downstream Raf, and hence, blocked the MAPK growth pathway in a way not achieved before. Since each cancer phenotype features a different ratio of oncogenic KRAS subtypes, the authors postulated that this allosteric inhibition will not be limited to certain cancer subtypes as it unexclusively targets wild-type and mutant (KRAS^G12C/D^ and KRAS^Q61H^) GTP-bound KRAS subtypes and can be used across a range of tumor types as a first-line therapy. The study was conducted in four oral and four lung cancer cell lines, with the latter known to be aggressive in nature, and features frequent KRAS mutation. This provides promise to the potential applicability of compound 11 in the treatment of other cancers with KRAS mutations, such as PDAC. Additionally, phosphorylated AKT levels were slightly decreased upon compound 11 treatment and indicate a suppressive interaction with the AKT pathway. This feature seems very important as it demonstrates an advantage over the previous studies mentioned in this article [24,28,29], which suffered from increased AKT pathway activation upon KRAS inhibition. 

Recently, another novel compound MRTX849, developed by Mirati Therapeutics [41], has entered clinical trials as a selective covalent KRAS^G12C^ inhibitor. MRTX849 is structurally and functionally similar to AMG 510 in the way that it binds to KRAS and has also showed promising potency in a range of cell lines, including lung and pancreatic cancers. In fact, the compound was able to exhibit significant antitumor activity as a monotherapy in these cell lines. Among those examined, the IC_50_ value of three non-mutant KRAS cell lines were significantly higher than the mutant cells. Thus, similar to AMG 510, this suggests that the presence of KRAS^G12C^ is required for MRTX849 activity. Phase I clinical trials of the single agent were associated with a partial response in two patients with advanced cancers (lung and colon). Further, recent results from this clinical trial have shown a partial response in four patients and stable disease in the rest of the evaluable patients (*n* = 12) at the time of analysis [26]. These preliminary observations with KRAS inhibitors provide hope for their use in PDAC. 

## 3. Drug Delivery to PDAC

As well as a high rate of resistance to drug treatment, PDAC has the added complexity of impaired tissue drug delivery issues [42,43]. As pancreatic tumors are often late-stage solid tumors, they contain a thick stromal compartment, which can be difficult to perfuse [43]. These tumors also tend to adapt to the stressful microenvironment with a low blood supply and become less reliant on it [43]. These features provide a major barrier for any kind of drug since less blood flow to the cells means a lesser chance of the drug reaching the target tumor site, and even if the compound enters the tissue through the semi-permeable neovascular membranes, the thick stroma prevents it from reaching the core tumor mass [43]. Not only is the actual delivery process difficult, but drugs like gemcitabine also suffer from biochemical and physiological issues, such as a very short plasma half-life and fast cellular degradation [44,45]. The combination of these features adds another level of complexity to PDAC drug treatment.

An emerging cooperation between nanoparticles and chemotherapy that has been extensively explored involves an anti-cancer compound that can piggy-back onto a nanoparticle for enhanced entry to the tumor site [46]. Nanoparticles are biocompatible and non-toxic molecules less than 50 nm in size that either entrap, adsorb, or attach to the active compound [46]. In a broad-scale review, Birhanu et al. [47] recently highlighted the prospects of nanotechnology in PDAC specializing in gemcitabine administration. The most successful nanoparticle variants were chitosan, gold, and albumin bound to gemcitabine, all of which were able to potentiate the anti-cancer efficacy of gemcitabine [47]. Gemcitabine combination with paclitaxel bound to albumin (nab-paclitaxel) is a common currently used chemotherapeutic regimen in PDAC treatment. Binding paclitaxel to 130-nm albumin particles allows it to cross the endothelial cell barrier more efficiently, allowing the administration of a higher dose while significantly enhancing the antitumor effects in combination with gemcitabine [5,48]. 

Other potential methods of tackling the tumor microenvironment are anti-fibrotic drugs. A dense stromal compartment is commonly found surrounding the core of the pancreatic tumor and, as mentioned previously, can act as a barrier against therapeutic entry and influence tumor progression as a supporting matrix [43]. This layer is formed from a diverse range of cell types that have been recruited by the tumor to assist in their survival [49]. The majority of the stroma, however, consists of fibroblasts, which are overactivated and cause an increase in the extracellular matrix (ECM) deposition [49]. This forms a tight ECM, which constricts nerve endings and local capillaries [49]. Methods of targeting fibroblasts have been investigated and found success by inhibiting the fibroblast activation protein (FAP), the driving force in ECM production [50,51]. Since fibroblasts have a relatively low activity under normal conditions and the PDAC stroma is highly active, antifibrotic drugs have displayed selectivity towards the PDAC stroma. An anti-fibrotic compound called halofuginone displayed in vitro and in vivo effectiveness against PDAC, which resulted in stromal breakdown, immune cell infiltration, and necrotic effects on cancer cells [52]. An alternative targeting method by Teichgräber et al. [53] used a human antibody, ESC11, and was able to fully deplete FAP to reduce neoplastic cells’ motility and migration. As the stroma is a major part of the pancreatic tumor’s composition, reducing the outer mass will allow for chemotherapeutics to penetrate the tumor more effectively and suppress the supporting cells.

Most anti-cancer agents are designed via the targeting of a critical core mechanism, resulting in potent anti-cancer activity under pre-clinical settings, but they usually tend to struggle in clinical trials from delivery and bioavailability issues. The prospect of nanoparticles and anti-fibrotic drugs could be an answer to the struggle against underwhelming PDAC therapy due to these critical clinical issues.

## 4. Conclusions

There is a big void to be filled in frontline PDAC therapy; here, “targeted” therapies and KRAS inhibitors appear to be very promising. Combining the antitumor effects from innovative new KRAS inhibitors like AMG 510 with other agents, nanoparticles, or other auxiliary processes that can overcome the PDAC biochemical and tissue delivery issues holds hope for a new therapeutic way forward in PDAC.
